# Health Tract, No. 21

**Published:** 1860-04

**Authors:** 


HEALTH TRACT, No. 21.

### ATTENTION TO THE FEET.

It is utterly impossible to get well or keep well, unless the feet are kept dry and warm all the time. If they are for the most part cold, there is cough or sore throat, or hoarseness, or sick headache, or some other annoyance.

If cold and dry, the feet should be soaked in hot water for ten minutes every night, and when wiped and dried, rub into them well, ten or fifteen drops of sweet oil ; do this patiently with the hands, rubbing the oil into the soles of the feet particularly.

On getting up in the morning, dip both feet at once into water, as cold as the air of the room, half ankle deep, for a minute in Summer ; half a minute or less in Winter, rubbing one foot with the other, then wipe dry, and if convenient, hold them to the fire, rubbing them with the hand until perfectly dry and warm in every part.

If the feet are damp and cold, attend only to the morning washings, but always at night remove the stockings, and hold the feet to the fire, rubbing them with the hands for fifteen minutes, and get immediately into bed.

Under any circumstances, as often as the feet are cold enough to attract attention, draw off the stockings, and hold them to the fire ; if the feet are much inclined to dampness, put on a pair of dry stockings, leaving the damp ones before the fire to be ready for another change.

Some person's feet are more comfortable, even in Winter, in cotton, others in woolen stockings. Each must be guided by his own feelings. Sometimes two pair of thin stockings keep the feet warmer, than one pair which is thicker than both. The thin pair may be of the same or of different materials, and that which is best next the foot, should be determined by the feelings of the person.

Sometimes the feet are rendered more comfortable by basting half an inch thickness of curled hair on a piece of thick cloth, slipping this into the stocking, with the hair next the skin, to be removed at night, and placed before the fire to be perfectly dried by morning.

Persons who walk a great deal during the day, should, on coming home for the night, remove their shoes and stockings, hold the feet to the fire until perfectly dry ; put on a dry pair, and wear slippers for the remainder of the evening.

Boots and gaiters keep the feet damp, cold and unclean, by preventing the escape of that insensible perspiration which is always escaping from a healthy foot, and condensing it ; hence the old-fashioned low shoe is best for health.

